# Cassava whitefly species in eastern Nigeria and the threat of vector-borne pandemics from East and Central Africa

**DOI:** 10.1371/journal.pone.0232616

**Published:** 2020-05-07

**Authors:** Joachim Nwezeobi, Onyeyirichi Onyegbule, Chukwuemeka Nkere, Joseph Onyeka, Sharon van Brunschot, Susan Seal, John Colvin

**Affiliations:** 1 Natural Resources Institute, University of Greenwich, Chatham Maritime, Kent, United Kingdom; 2 National Root Crops Research Institute, Umudike, Abia, Nigeria; 3 School of Biological Sciences, The University of Queensland, St. Lucia, Queensland, Australia; Harran University, TURKEY

## Abstract

*Bemisia tabaci* (*sensu latu*) is a group of >40 highly cryptic whitefly species that are of global agricultural importance, both as crop pests and plant-virus vectors. Two devastating cassava diseases in East and Central Africa are spread by abundant populations of one of these species termed Sub-Saharan Africa 1 (SSA1). There is a substantive risk that these whitefly-borne pandemics will continue to spread westwards and disrupt cassava production for millions of smallholder farmers in West Africa. We report here, therefore, the first comprehensive survey of cassava *B*. *tabaci* in eastern Nigeria, a West African region likely to be the first affected by the arrival of these whitefly-borne pandemics. We found one haplotype comprising 32 individuals with 100% identical mtCO1 sequence to the East African SSA1 populations (previously termed SSA1-SG1) and 19 mtCO1 haplotypes of Sub-Saharan Africa 3 (SSA3), the latter being the most prevalent and widely distributed *B*. *tabaci* species in eastern Nigeria. A more divergent SSA1 mtCO1 sequence (previously termed SSA1-SG5) was also identified in the region, as were mtCO1 sequences identifying the presence of the MED ASL *B*. *tabaci* species and *Bemisia afer*. Although *B*. *tabaci* SSA1 was found in eastern Nigeria, they were not present in the high abundances associated with the cassava mosaic (CMD) and cassava brown streak disease (CBSD) pandemics of East and Central Africa. Also, no severe CMD or any CBSD symptoms were found in the region.

## Introduction

*Bemisia tabaci* Gennadius (*sensu latu*) is a group of more than 40 morphologically indistinguishable (cryptic) putative whitefly species [1–10]. Partial sequences of the mitochondrial cytochrome oxidase 1 (mtCO1) gene have been used as the DNA barcode to identify and delimit these species [1], but it is increasingly accepted that an integrative approach to discovering the full diversity of species within *Bemisia tabaci* is required [2,3,11]. *B*. *tabaci* species cause considerable economic damage to crops through, (i) direct leaf damage, (ii) promotion of saprophytic fungal growth by their excretion of honeydew on to plant leaves and (iii) the transmission of more than 200 plant viruses [4,12], which include cassava mosaic viruses (CMVs) [13] and cassava brown streak viruses (CBSVs) [14]. Cassava (*Manihot esculenta*, Crantz) is an important food security crop in sub-Saharan Africa (SSA) because it is resilient to harsh climatic conditions and is a rich source of carbohydrates [15,16]. Cassava production in sub-Saharan Africa (SSA), however, has been reduced and threatened by the emergence and rapid spread of whitefly-borne diseases caused by CMVs and CBSVs [17–20].

A pandemic of severe cassava mosaic disease (CMD) began in Uganda in the 1980s [21] and quickly spread throughout East Africa (EA) and into Central African countries [22]. Cassava brown streak disease (CBSD) is causing another growing pandemic in EA and has spread westwards from its original geographic range in the coastal regions and surrounding the Great Lakes in Central Africa [23–25]. Both pandemics are associated with high populations of African cassava *B*. *tabaci* and the SSA1 (also referred to as SSA1-SG1) population, in particular [5,6]. There is a real risk and concern that these pandemics, driven by abundant whitefly-vector populations, will continue to spread westwards, although there is no evidence yet that they have reached West Africa [26,27].

In comparison to the information documenting the *B*. *tabaci* species diversity in East Africa, the situation in West Africa is relatively unknown. Of the sixteen West African countries, *B*. *tabaci* survey reports are available only for Burkina Faso, Benin, Côte d'Ivoire, Liberia, Mali, Nigeria, Sierra Leone and Togo [28–32]. Leuschner [33], at the International Institute of Tropical Agriculture (IITA), Ibadan, Nigeria, conducted the first recorded survey of whitefly populations in West Africa from 1976–1977 and reported annual and seasonal variations in whitefly population densities. Fargette *et al*. [34] then surveyed whiteflies on cassava in Cote d’Ivoire and in 1995 a further study of *B*. *tabaci* populations in Côte d'Ivoire was published [7], where the number of whitefly adults caught on sticky traps situated in and around cassava plots was investigated. Additional whitefly surveys were reported for Ghana [35], Benin [36] and Nigeria [37,38] a decade later. In these earlier studies, however, morphological differences alone were used to identify the whitefly species collected. The reliance on classical taxonomic differences (morphological) for species identification in these studies, therefore, meant that it is impossible to interpret the data in terms of the current *B*. *tabaci* species naming system, which is based on partial mtCO1 gene-sequence divergence [1].

Burban *et al*. [39] conducted the first molecular identification of whitefly species in West Africa. The study identified the cassava and the okra (*Abelmoschus esculentus* (L.) Moench) biotypes among sympatric whitefly populations in central and southern Côte d'Ivoire, which differed in their iso-enzyme electrophoresis patterns and host-plant preferences. The okra biotype was collected from all of the plant hosts they surveyed, except cassava. The cassava biotype, however, was only present on cassava and eggplant (*Solanum melongena* L.) [39]. Omondi *et al*. [40] also identified the cassava and okra biotypes in Ghana using the same molecular methods and their results confirmed that the okra and cassava biotypes had the highest preferences for okra and cassava, respectively. Until 2006, therefore, all the whitefly surveys conducted in West Africa used either morphological characteristics or electrophoresis patterns for identifying whitefly populations.

De La Rúa *et al*. [41] was the first West African study to use mtCO1 sequences to investigate the diversity of *B*. *tabaci* there. Out of 54 whitefly specimens used in the study, 20 originated from Ghana, Mali and Nigeria and the remainder came from Italy, Spain and Turkey [41]. They identified three *B*. *tabaci* putative populations among specimens obtained from sweet potato (*Ipomoea batata* (L.) Lam), okra, cassava and cowpea (*Vigna unguiculata* (L) Walp). The MED species, referred to as biotype J in the study, were collected from okra and sweet potato in Ghana and cowpea in Nigeria. The study also identified the SSA2 species, which they referred to as Sub-Saharan VI, on cassava in Mali and Nigeria. The third population, SSA1-SG5, named “Sub-Saharan II” in the study, was found in Ghana and was only collected on cassava [41]. Sartor *et al*. [28] also conducted similar surveys in Italy, Spain and the West African country of Mali. In Mali, they identified “Sub-Saharan VI” on cassava only and the MED species (biotypes Q and J) on several host plants, but not on cassava. There was no accession number associated with “Sub-Saharan VI” in their report, so its identity remains uncertain.

Gnankine *et al*. [29] identified *B*. *tabaci* populations in Burkina Faso that were named Q1, Q3 and African silver-leafing (ASL) based on their mtCO1 sequences. They also described Q1, ASL, African non-silver-Leafing 1 (AnSL1) and African non-silver-leafing 2 (AnSL2) populations from Benin and Togo. The first three ‘biotypes’ (Q1, Q3 and ASL) belong to the Mediterranean (MED) putative species group, while the AnSL1 and AnSL2 ‘biotypes’ belong to the SSA3 putative species. The MED species were found on sweet potato, tomato, okra, cotton and tobacco. The AnSL1 and AnSL2 biotypes (SSA3) were the only biotypes reported on cassava in the study [29]. More recently, two surveys have been reported that detail the distribution of whiteflies in south-western [31,42] and north-central Nigeria [42]. The surveys in the region reported up to 35 whitefly species including *B*. *tabaci* and *B*. *afer*. The reports, however, only identified the whiteflies based on their morphology and did not use mtCO1 markers to identify the members of the *B*. *tabaci* species complex present in the region.

Ghosh *et al*. [8], however, did obtain mtCO1 sequences for the cassava whiteflies they characterised from Nigeria, Tanzania, Malawi and Uganda. In Nigeria, they identified three *B*. *tabaci* populations, which were SSA1-SG1, SSA3 and SSA1-SG5. SSA3 individuals were found only in eastern Nigeria, while the SSA1-SG1 and SSA1-SG5 were collected from cassava plants in western Nigeria. So far, studies using mtCO1 for *B*. *tabaci* classification in West Africa have reported five main *B*. *tabaci* populations in the region, including (i) MED, (ii) SSA1-SG1, (iii) SSA1-SG5, (iv) SSA2 and (v) SSA3. Two recent reports have, however, attempted to reclassify some members of the *B*. *tabaci* sub-Saharan (SSA) based on single nucleotide polymorphism markers generated using NextRAD sequencing [32,43]. The studies reclassified both the Nigerian population of SSA1-SG5 and Cameroon population of SSA1-SG1 as SSA-WA and also the East and Central African populations of SSA1-SG1 as SSA-ECA. The reclassification was, however, not complemented by any biological evidence of speciation, such as mating compatibility, among the populations. A genome was later generated for the SSA-ECA population [32].

The research presented here was carried out in eastern Nigeria because this West African region is likely to be the first affected by the arrival of *B*. *tabaci* SSA1 (‘-SG1’)-borne cassava-disease epidemics spreading from East and Central Africa. The pandemics have spread from their origin in East Africa and in the past decade spread to Central Africa [26,27]. Little is known, however, about the genetic diversity of *B*. *tabaci* populations on cassava and other host plants in eastern Nigeria, or their distributions in the different agro-ecological zones. The survey-data presented here, therefore, provide a baseline data-set against which future changes to the whitefly populations and disease incidences can be assessed.

## Materials and methods

### Survey region and whitefly collection

The survey region consisted of ten eastern Nigerian states, one of which is bordered with Cameroon to the east. These states were: (i) Abia, (ii) Akwa Ibom, (iii) Anambra, (iv) Ebonyi, (v) Edo, (vi) Enugu, (vii) Cross-river, (viii) Delta, (ix) Imo and (x) Rivers, which cover a total area of *c*. 120,000 km^2^. The survey region included sub-humid and humid-tropic agro-ecological zones and was bordered in the north by Kogi and Benue states, to the west by the Atlantic Ocean and Ondo state and by the Atlantic Ocean in the south. Anambra, Ebonyi, Edo, Enugu and Imo states lay within the sub-humid tropics zone, with an annual rainfall of *c*. 1,682–2,217 mm. The rainy season there usually begins in mid-March, peaks in June-July and ends in early December [44]. Cross River, Delta and Abia lie between the sub-humid tropic and humid-tropic agro-ecological zones, while Akwa Ibom and Rivers were situated entirely in the humid-tropic zone. The humid tropic region has an annual rainfall of *c*. 1,700–4,700 mm and experiences rainfall throughout the year except for January and February. In general, rainfall increased with decreasing distance from the Atlantic Ocean [44].

Two surveys were carried out in 2015. The first survey occurred in March-April 2015, at the start of the rainy season, when only a few farms had young crops and others remained fallow. Whiteflies were collected from cassava, cucumber (*Cucumis sativus*), castor bean (*Ricinus communis*), morning glory (*Ipomoea purpurea*) and okra. This weed (*I*. *purpurea*) was chosen for collecting whiteflies, because it was observed to grow near or within cassava fields. The number of whiteflies on the top five leaves of each plant was counted. The second survey was conducted from March-December 2015, by the staff of the National Roots Crops Research Institute (NRCRI). The latter survey focussed primarily on collecting whitefly from cassava plants. Both surveys, however, sampled whitefly adults from plants selected at random from each field, using a mouth aspirator (Watkins and Doncaster pooter). Altogether, 119 plants in 119 locations were selected randomly and whitefly samples collected. The whiteflies were then transferred from the aspirator to 1.5 ml Eppendorf tubes and preserved in 70% ethanol. The location for each field was recorded using a GT-120 igotU GPS receiver. The GPS coordinates were entered into the QGIS software (v3.4) and used to produce a map of the sampled locations. The HarvestChoice sub-Saharan agroecological zones plugin was used to overlay the agro-ecological zones on the map [45].

### Ethics statement

This study did not involve human participants nor tissues, embryos and vertebrate animals and hence did not require specific permits. Whiteflies are invertebrate insects and, according to the IUCN criteria, are not considered as endangered or protected species. The whitefly sampling was conducted on private lands and for each location, permission was obtained from the land owner for the whitefly survey and collection.

### Whitefly DNA extraction

One hundred and nineteen locations were sampled in this study and two whitefly samples were selected randomly from each field and genotyped separately. Single whitefly adults were randomly chosen from each of the 1.5 ml Eppendorf tubes containing 70% ethanol, using a sterilized entomological pin. Each whitefly was transferred to a clean 1.5 ml Eppendorf tube. To extract insect DNA, 50 μl of 10% Chelex was added to the tube and the whitefly was crushed using a plastic pestle until a clear homogenised mixture was obtained. The mixture was then incubated at 56°C for 20 minutes and further incubated at 100°C for five minutes. The mixture was then centrifuged at 13,500 g for five minutes using the Eppendorf Centrifuge 5424 R and the supernatant was collected and stored at -20°C until use.

### Partial mitochondrial CO1 gene amplification and gel electrophoresis

Partial mtCO1 gene sequences were amplified using 2 μL of the extracted DNA. Two sets of primers, with different degrees of sensitivity, were used to amplify the mtCO1 fragments, which were the: (i) African specific 2195Bt (5’-TGRTTTTTTGGTCATCCRGAAGT-3’) and C012/Bt-sh2 (5’-TTTACTGCACTTTCTGCC-3’) primers [6] and (ii) generic CI-J-2195 (5’- TTGATTTTTTGGTCATCCAGAAGT -3’) and TL2-N-3014 (TCCAATGCACTAATCTGCCATATTA -3’) primers [46]. Polymerase chain reaction (PCR) was run on an Applied Biosystems 2720 thermal cycler for 35 cycles. The PCR program conditions were set at: (i) initial denaturation at 94°C for 2 minutes, (ii) denaturation at 94°C for 20 seconds, (iii) annealing at 52°C for 30 seconds, (iv) extension at 72°C for one minute and (v) final extension at 72°C for five minutes. Agarose gel electrophoresis (1% w/v in 0.5 X Tris/borate/EDTA buffer) was used to confirm PCR amplification. The GeneJET PCR purification kit (Thermo Scientific) was used to purify amplicons of interest as described by the kit manufacturer. After purification, amplicons were sent to Eurofins Genomics for Sanger sequencing.

### Phylogenetic analysis

The partial mtCO1 sequences obtained from sequencing two randomly selected whiteflies from each of the 119 locations were first screened using quality checks in Geneious (v10.2.6) [47] and those failing to meet the following criteria were discarded. First, the sequence chromatograms were manually checked and translated using the invertebrate mitochondrial genetic code. Poor quality sequences and sequences containing stop codons were excluded and the sequencing process repeated for these samples. Two hundred and thirty-eight contig sequences were assembled from sequence data that passed the quality control and were trimmed to 657 bp and aligned with 691 *B*. *tabaci* mtCO1 reference sequences downloaded directly from GenBank. The resulting alignment was used to plot an initial phylogenetic tree that was used to identify the whitefly genetic groups, using the FastTree program in Geneious (v10.2.6) [47,48].

Twenty-nine unique haplotypes were identified in the 238 new sequences, which were extracted with Geneious 10.2.6 and used to generate the main phylogenetic tree. These unique sequences were combined with 18 selected reference sequences and aligned. A mtCO1 sequence of the spiralling whitefly *Aleurodicus dispersus* Russell (GenBank code: AJ748380) was used as an outgroup for the analysis. The best model of nucleotide sequence evolution was evaluated using jModelTest (v2.1.10) [49]. MrBayes (v3.2.6) was run using the nexus-formatted aligned sequences and the Generalised time-reversible (GTR) model [50,51] and Gamma distribution with invariant sites (G+I). MrBayes (v3.2.6) was run with four chains for 50 million generations and the trees were sampled every 1,000 generations. The consensus tree (.nex.con.tre) file was used to view the phylogenetic tree and the posterior probabilities of the branches using FigTree (v1.4.3) [52]. The tree was further annotated using Adobe Illustrator (v22.1). The 29 unique haplotype sequences were submitted to the GenBank sequence database and were each assigned unique accession numbers. The sequence ID and their accession numbers (in brackets) are as follows: NRI_108a (MN164753), NRI_104 (MN164754), NRI_098b (MN164755), NRI_088b (MN164756), NRI_088a (MN164757), NRI_087a (MN164758), NRI_067a (MN164759), NRI_051b (MN164760), NRI_048a (MN164761), NRI_035b (MN164762), NRI_030a (MN164763), NRI_010b (MN164764), NRCRI_86b (MN164765), NRCRI_86a (MN164766), NRCRI_81a (MN164767), NRCRI_75a (MN164768), NRCRI_69b (MN164769), NRCRI_69a (MN164770), NRCRI_066b (MN164771), NRCRI_54a (MN164772), NRCRI_36b (MN164773), NRCRI_33b (MN164774), NRCRI_25a (MN164775), NRCRI_17b (MN164776), NRCRI_13a (MN164777), NRCRI_12a (MN164778), NRCRI_08a (MN164779), NRCRI_07a (MN164780) and NRCRI_05b (MN164781).

### Genetic network and data analysis

The 29 unique haplotypes were used to generate a minimum spanning tree. The alignment file (.nex) obtained from Geneious was used to build the minimum spanning tree using Phyloviz [53], to identify the relationships and mutational distances between the different haplotypes.

After the random selection and testing of two whitefly samples per field, a two-sided Fisher test and the chi-square test were carried out on the number of different populations identified in the two samples to ascertain whether or not more sampling was needed to capture the entire diversity of the genetic groups present in the survey collections. The protocols for whitefly DNA extraction, mtCO1 amplification, phylogenetic analysis and genetic network analysis have been submitted in the protocols.io repository and has a doi of dx.doi.org/10.17504/protocols.io.bd6gi9bw.

## Results

### Phylogenetic and statistical analysis

**Fig 1:**
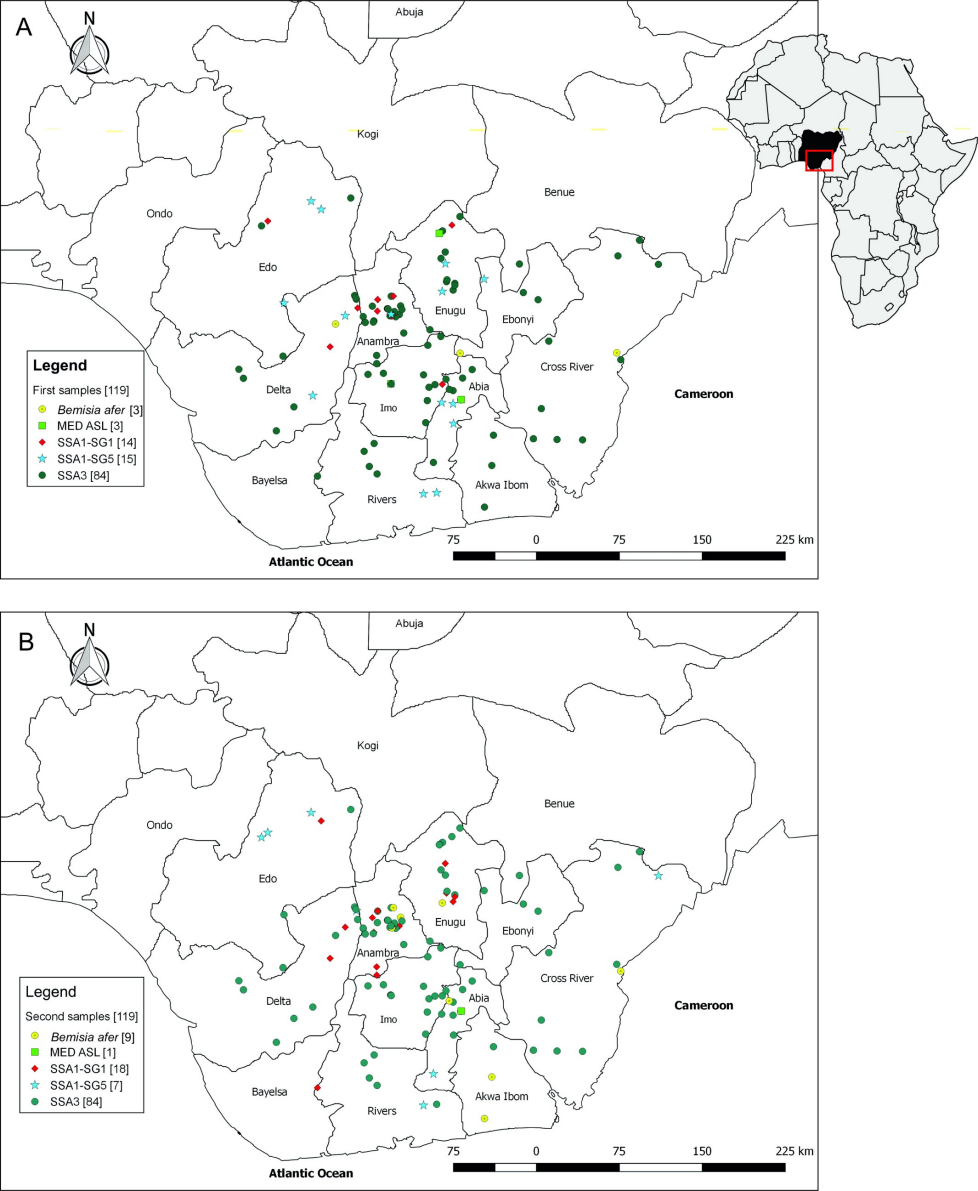
The locations of fields where whiteflies were collected in eastern Nigeria. Five different symbols were used to represent the five populations identified in the first (A) and second (B) samples. The colours and shapes represent the following: (i) yellow circles for *B*. *afer*, (ii) green squares represent MED ASL, (iii) red rhombus are SSA1-SG1, (iv) light blue stars are SSA1-SG5 and (v) green circles represent SSA3.

Total DNAs were extracted and partial mtCO1 genes sequenced successfully for 238 whiteflies, representing two individual whiteflies from each of 119 locations. Phylogenetic analyses conducted on sequences from the first sampling showed that the SSA3 putative species was the most common, represented by 84 (70.6%) of the total number of adults in the first sample (Fig 1A). The other *B*. *tabaci* mtCO1 groups found were: 29 SSA1 sequences (14 of SSA1-SG1 (11.8%), 15 of SSA1-SG5 (12.6%)) and three MED ASL (2.5%). Three of the adults (2.5% of the sample set), were *Bemisia afer* (Prisner & Hosny). The results obtained from the second sample were similar to the first, with minor variations. SSA3 was the predominant whitefly population in the second sample (84 individuals (70.6%)) (Fig 1B). The individuals sequenced were SSA3 in 61 of the survey sites. In the other 23 sites, there was a combination of SSA3 and either of *B*. *afer*, SSA1-SG1 or SSA1-SG5 populations. The other genetic groups identified included 18 of SSA1-SG1, seven of SSA1-SG5 and only one MED ASL, which represented 15.1%, 5.9% and 0.8%, respectively, of the second set of samples. There were also nine (7.6%) *B*. *afer* identified in the second sample, compared to only three identified in the first sample. The two-sided Fisher test showed that the sample results were not significantly different (*P* = 0.113). The less stringent chi-square test on the two samples also yielded a non-significant *P*-value of 0.116.

**Fig 2:**
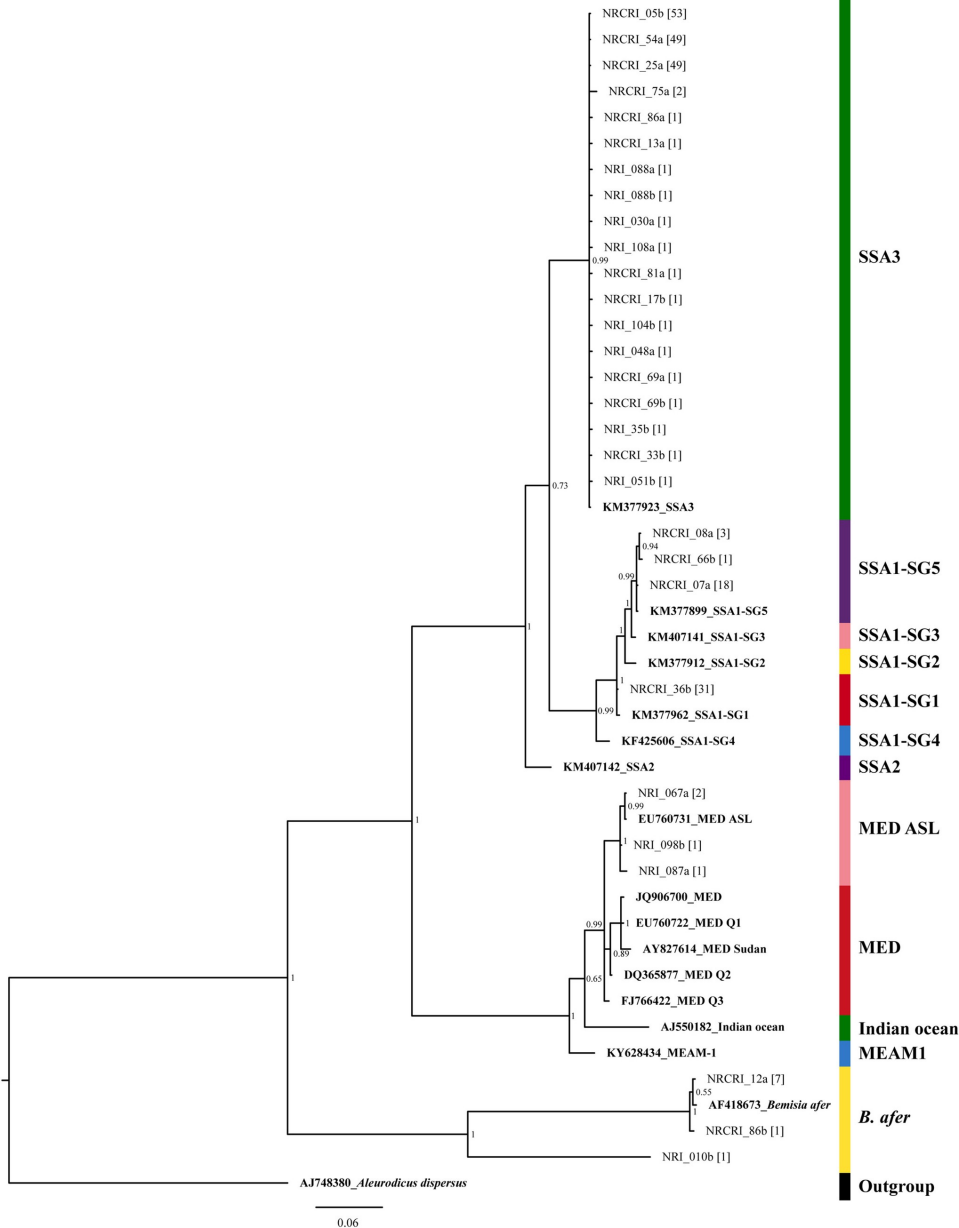
The phylogenetic tree showing the relationships between 29 unique partial mtCO1 haplotypes and 18 reference partial mtCO1 sequences. The different coloured bars represent the following: (i) green represents SSA3 and Indian ocean, (ii) purple represents SSA1-SG5 and SSA2, (iii) pink represents SSA1-SG3 and MED ASL, (iv) yellow represents SSA1-SG2 and *B*. *afer*, (v) red represents SSA1-SG1 and MED, (vi) blue represents SSA1-SG4 and MEAM1 and (vii) black represents the outgroup, *Aleurodicus dispersus*. The number of individuals in each unique haplotype is enclosed in a square bracket beside the name of one representative from the haplotype. The reference sequences appear in bold fonts and have their accession number preceding them.

The phylogenetic tree built using the 29 unique haplotypes obtained from the 248 sequences derived in this study (Fig 2) showed that there were 19 unique haplotypes for the SSA3 species, which is the highest number of unique haplotypes amongst the five genetic groups identified. Three of the 19 SSA3 haplotypes had more than 47 individuals each, one haplotype had only two individuals and the other 15 haplotypes had just a single individual each. The SSA1-SG1 surprisingly had only a single unique haplotype with 32 individuals in the haplotype, even though these were collected from five different states. The same mtCO1 haplotype of SSA1-SG1 has been reported in three east African countries (Kenya, Tanzania, Uganda) and three central African countries (Burundi, Central African Republic and the Democratic Republic of Congo) (Table 1). The SSA1-SG5, MED ASL and *B*. *afer* each had three unique haplotypes.

**Table 1:** Select list of haplotypes 100% similar to the SSA1-SG1_Ng identified in this study.

S/N	Hit Accession	Percent Identity	Sequence length	Max Score	Host plant	Country
1	KF425589.1	100	657	1214	Cassava	Burundi
2	LT707470.1	100	504	928	Cassava	Central African Republic
3	HE573754.1	100	657	1214	Cassava	Democratic Republic of Congo
4	JQ286429.2	100	657	1214	Cassava	Kenya
5	DQ130057.1	100	657	1214	Cassava	Kenya
6	KY523852.1	100	654	1208	Cassava	Kenya
7	JQ286479.2	100	657	1214	Cassava	Tanzania
8	KX570785.1	100	657	1214	Muwugula	Uganda
9	AM040604.1	100	657	1214	Cassava	Uganda
10	AY903517.1	100	657	1214	Tomato	Uganda
11	AY903515.1	100	657	1214	Cassava	Uganda
12	AY903497.1	100	657	1214	Okra	Uganda
13	AY903477.1	100	657	1214	Jatropha	Uganda
14	AY057209.1	100	657	1214	Cassava	Uganda
15	KM377917.1	100	657	1214	Cassava	Uganda

### Minimum spanning network analysis

**Fig 3:**
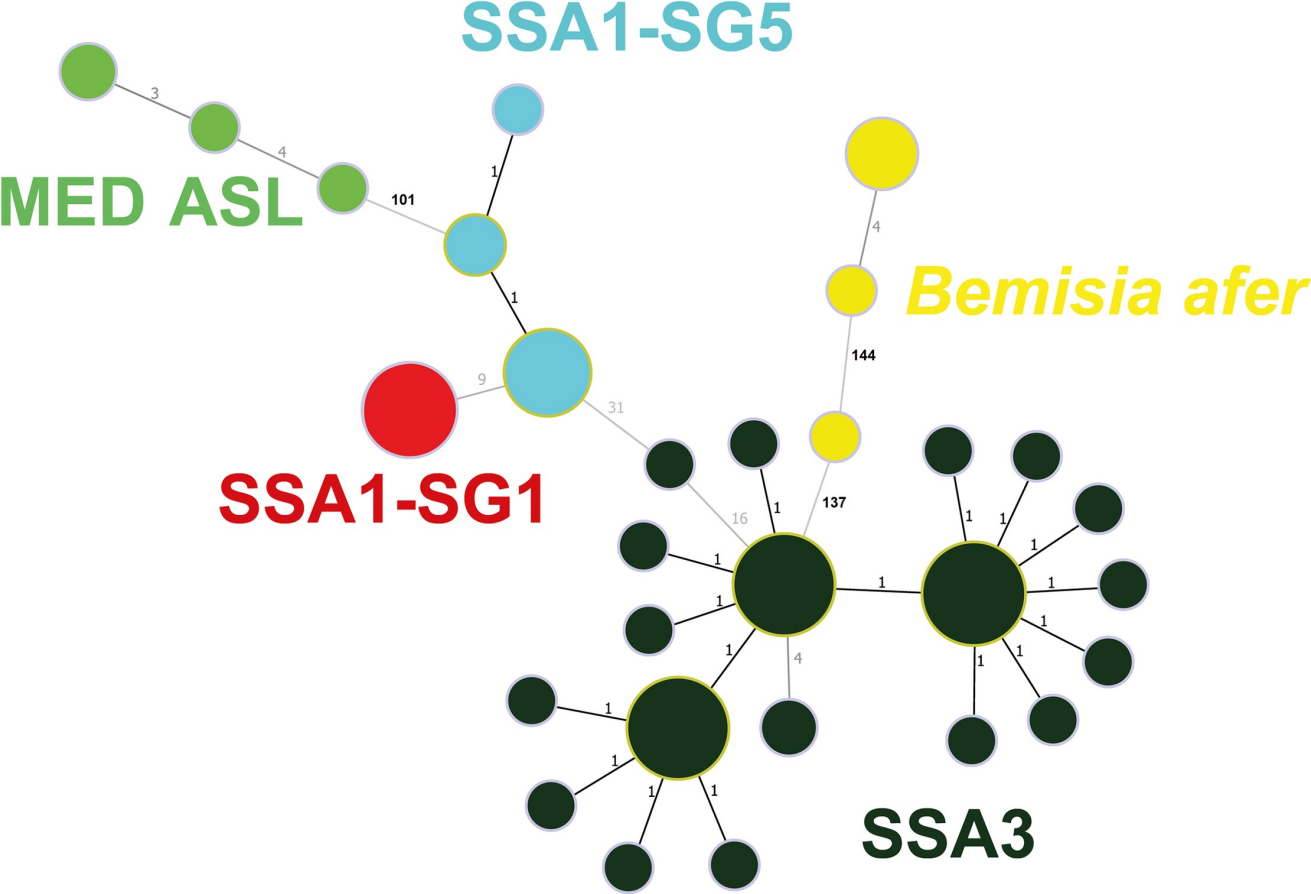
Minimum spanning network showing the evolutionary relationships between 29 unique haplotypes across five whitefly populations analysed using the mtCOI sequences generated from 238 whitefly samples collected in eastern Nigeria. The populations include *Bemisia afer*, MED ASL, SSA1-SG1, SSA1-SG5 and SSA3. The mutational steps between haplotypes are shown as numbers beside the lines. The circle sizes loosely represent the number of individuals represented in each haplotype.

The Minimum Spanning Tree (MST) showed the number of unique haplotypes amongst *B*. *afer*, MED ASL, SSA1-SG1, SSA1-SG5 and SSA3, to be 3, 3, 1, 3 and 19, respectively (Fig 3). Circles represent the unique haplotypes and their sizes are relative to the number of individuals possessing that haplotype. The SSA1-SG5 population, for example, was separated from MED ASL by a minimum of 101 mutational steps. The three SSA populations had less than 35 predicted mutational steps separating them. As was expected, the *B*. *afer* haplotype group was separated from the rest of the *B*. *tabaci* populations by the greatest distance of a minimum of 137 mutational steps. Of interest was the greater diversity within the *B*. *afer* haplotype group, compared to that within the *B*. *tabaci* species group. One of the unique *B*. *afer* haplotypes differed from another by up to 144 mutational steps, which is higher than the number of mutational steps that separated *B*. *afer* from all the *B*. *tabaci* (*sensu latu*) individuals. A nucleotide blast search of the three unique *B*. *afer* sequences revealed that NRCRI_12a and NRCRI_86b produced top hits (>99% sequence similarity) to sequences identified as *B*. *afer*. The sequence of the third *B*. *afer* (NRI_010b) which differed markedly from the others produced a top hit with another *B*. *afer* sequences [54] but with only *c* 80% similarity to the sequence.

### Distribution of *B*. *tabaci* genetic groups according to agro-ecological zones and host plants

**Fig 4:**
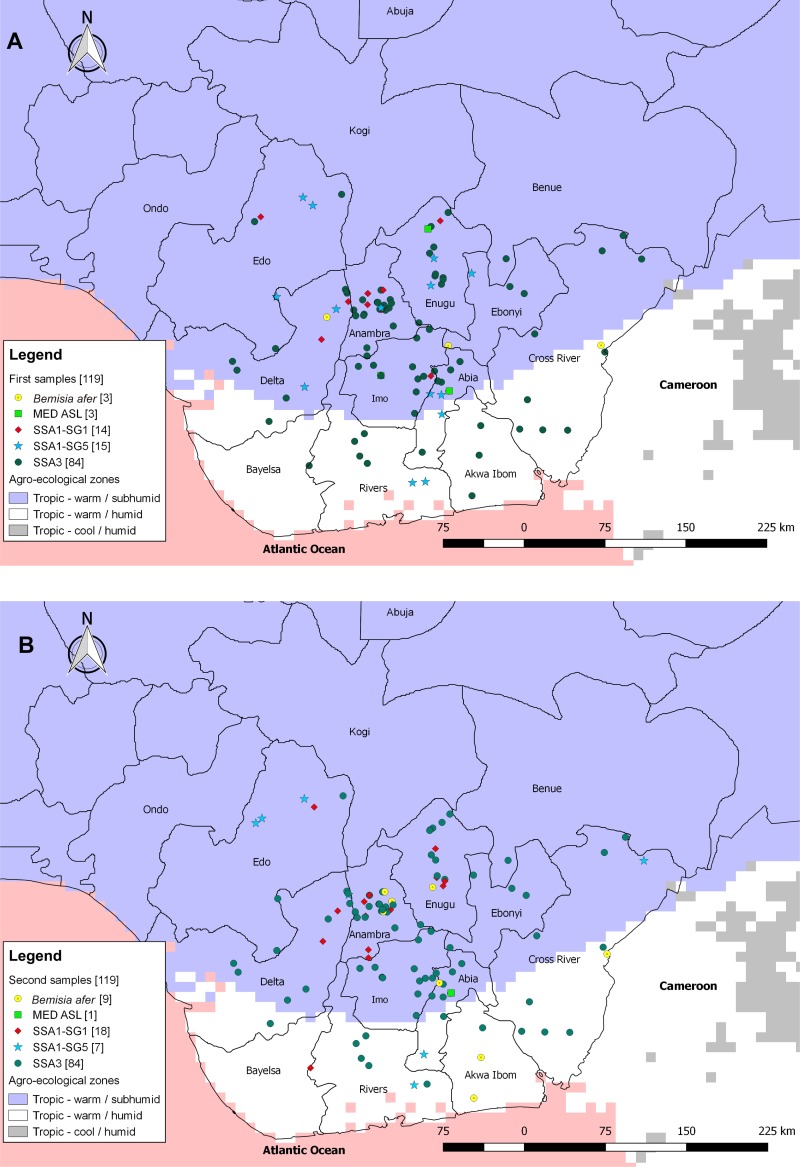
Cut-out maps of eastern Nigeria showing the distribution of four *B*. *tabaci* populations and *B*. *afer* identified in the first (A) and second (B) samples across agro-ecological zones. The agro-ecological zones are coloured on the map. Indigo was used to represent the sub-humid area; white was used to represent the warm humid area and dark grey was used to represent the cool humid area.

A significant effect was observed in the distribution of the SSA1-SG1 and MED ASL populations across the two agro-ecological zones surveyed. In the first sampling, both populations (SSA1-SG1 and MED ASL) were only identified in the sub-humid tropics region (Fig 4A). The second sampling produced a highly similar distribution pattern to the first, except that one SSA1-SG1 population was found in the humid tropic region at the border between Bayelsa and Rivers states (Fig 4B). Minor variations in distribution across the two agro-ecological zones were also observed between the results from the two sampling efforts among the three populations, SSA3, SSA1-SG5 and *B*. *afer* (Fig 4). The three populations were, however, proportionately distributed across two agro-ecological zones for the two samplings.

Adults of all the five whitefly populations were found on cassava, with the most and least common, respectively, being 154 of SSA3 and one MED ASL. The rest of the host plants including cucumber, okra and castor bean had one or two different *B*. *tabaci* populations collected from them. Apart from MED ASL, the other four whitefly genetic groups were collected on *I*. *purpurea*. There were some differences in the species identified on the different plants for the two samples. In the first sample, the SSA3 was collected from all the host plants except cucumber. *B*. *afer*, SSA1-SG1 and SSA1-SG5 were only collected from cassava (Table 2A). The three MED ASL identified were collected from three of the host plants including cassava, cucumber and okra. In the second sample, SSA3 was present on all of the host plants including cucumber (Table 2B). The two subgroups of SSA1 and the *B*. *afer* were collected from cassava and *I*. *purpurea*. The only MED ASL individual identified in the second round of testing was collected on okra.

**Table 2:** Distribution of *B*. *tabaci* mtCO1 sequence populations on five host plants for the: (a) first and (b) second individuals sequenced per collection site. A 657 bp region of the mtCO1 was used for the species delimitation.

**a) First set of individuals**						
Host plants	*Bemisia afer*	MED ASL	SSA1-SG1	SSA1-SG5	SSA3	Number of samples
Cassava	3	1	14	15	76	109
Cucumber	-	1	-	-	-	1
Okra	-	1	-	-	2	3
Castor bean	-	-	-	-	1	1
Morning glory	-	-	-	-	5	5
Grand total	3	3	14	15	84	119
**b) Second set of individuals**						
Host plants	*Bemisia afer*	MED ASL	SSA1-SG1	SSA1-SG5	SSA3	Number of samples
Cassava	8	-	17	6	78	109
Cucumber	-	-	-	-	1	1
Okra	-	1	-	-	2	3
Castor bean	-	-	-	-	1	1
Morning glory	1	-	1	1	2	5
Grand total	9	1	18	7	84	119

## Discussion

### *B*. *tabaci* species distribution across eastern Nigeria

This report is the first extensive sampling and molecular identification of *B*. *tabaci* colonising cassava and other plant species in eastern Nigeria. The specimens were typed using the mtCO1 marker and the *B*. *tabaci* mtCO1 species groups identified were: (i) SSA3, (ii) SSA1 (-SG1 and -SG5), and (iii) MED (-ASL). *B*. *afer* also made up 5% of the 238 individuals sequenced. SSA3 *B*. *tabaci* were the most ubiquitous, being uniformly distributed across the sampled region, occurring in all ten states. SSA3 *B*. *tabaci* have not been reported from East Africa to date, but previous surveys have reported SSA3 from eastern Nigeria, Benin, Cameroon, Togo and Central Africa Republic [8,27,29,43].

The previous lack of research on SSA3 probably reflects its current non-pest status, so little is known about its virus transmission ability, mating compatibility with other *B*. *tabaci* populations, fecundity and response to insecticides. Although the SSA3 is ubiquitous across the region we surveyed, due to their low numbers, they do not appear to pose a significant threat to their cassava host-plants in terms of direct damage. We did, however, observe some CMD incidence in the field, but it was not as severe or widely distributed as the current CMD epidemic affecting cassava production in East and Central Africa (ECA). This suggests that that the SSA3 population may not be as an efficient vector of CMVs as SSA1-SG1. Also, efforts are now underway to explore the mating compatibility of SSA3 with other cassava whiteflies in sub-Saharan Africa, with a view to resolving the complicated phylogeny of the *B*. *tabaci* species complex and to discover if gene flow might be possible between the SSA3 *B*. *tabaci* and potential *B*. *tabaci* invaders from ECA, such asSSA1-SG1,.

The SSA1-SG5 genetic group was the least ubiquitous, but it still occurred in six out of the ten states and in the two agro-ecological zones surveyed. This population has also been reported on cassava in western Nigeria [8]. SSA1-SG5 also seemed to prefer cassava and was only collected once on *I*. *purpurea*, which was the same result as that for SSA1-SG1.

The MED-ASL is polyphagous but does not survive on cassava [28]. In this study, however, MED-ASL was collected once on cassava and cucumber and twice on okra. The scarcity of the MED-ASL in this survey was probably due to the bias towards collecting whitefly from cassava plants. A survey of other plants including eggplants and melons, which are both hosts of MED-ASL, would perhaps have revealed a more accurate distribution of this species. Also, the collection of a single MED-ASL adult on cassava was probably due to the individual resting on the cassava plant when it was collected, rather than it being a true host. This is because previous studies have shown that the MED-ASL adults die approximately two days after being given only cassava to feed on and that they do not lay viable eggs on this host-plant [28].

MED *B*. *tabaci* is made up of up to four sub-groups called Q1, Q2, Q3, and ASL based on their partial mtCO1 sequences [55]. Recent studies, however, have shown that Q1 and Q2 can mate and produce viable progeny, suggesting that the two subclades are the same biological species [3]. The MED Q populations, apart from the ASL, are globally invasive species and have been reported in several countries distant from their Mediterranean origins [56]. This survey, however, did not detect MED Q in eastern Nigeria. Our research was focused on cassava and it remains uncertain, therefore, if the Q population(s) of MED is present in eastern Nigeria. The occurrence of MED Q in eastern Nigeria would have severe consequences for whitefly control in the region, since the population previously reported in Burkina Faso was shown to have a knock-down resistance (kdr) mutation that made it resistant to the commonly used organophosphate and pyrethroid-based insecticides [57].

*B*. *afer* identification was not the focus of this research, but the species was collected because under field-collection conditions it can be difficult to distinguish it morphologically from *B*. *tabaci*. Similar to the results for *B*. *tabaci* (SSA3, SSA1-SG1 and SSA1-SG5), *B*. *afer* was found on cassava and only once on *I*. *purpurea*. Of the three unique haplotypes of *B*. *afer* identified in this study, the minimum spanning network analysis revealed that one haplotype differed from the others by up to 144 mutational steps. The scientific articles which reported these *B*. *afer* reference sequences did not conduct any morphological taxonomical identifications, prior to generating the mtCO1 sequence. The species, therefore, were most probably named based solely on their sequences clustering with other reported *B*. *afer* sequences. A blast search using the sequences of the distinct *B*. *afer* (NRI_010b) produced top hits with another *B*. *afer* (GenBank code: JX416161) identified by van Brunschot et al. [54]. Fortunately, the study reported that the *B*. *afer* whitefly were identified before crushing and amplification of mtCO1 sequences. The high nucleotide differences (>19%) between NRI_010b and the *B*. *afer* reported by van Brunschot et al. [54] may have two explanations. First, it may be that there is a rich diversity of cryptic species within the *B*. *afer* species that has not been studied yet. Second, the population is likely to be an unidentified species that clustered together with *B*. *afer*. Without comparative morphological data, we cannot be certain which explanation is correct.

*I*. *purpurea* is a common weed in eastern Nigeria and we collected four of the whitefly populations, except MED-ASL, on this plant species. We propose, therefore, that this weed may provide a resting place for whiteflies and may also serve as an alternative host-plant to the cassava whiteflies when cassava is unavailable. The original host(s) for the African cassava whiteflies is currently unknown, since cassava was only introduced into Africa from Latin America *c*. 400 years ago [58], whereas *B*. *tabaci* species have been evolving in the African continent for millions of years [6,9]. *I*. *purpurea* is not a native host for the whiteflies, since it also originated from the New World [59]. The paucity of samples collected from non-cassava plants and the collection of only adult plants, however, does not allow us to make substantive inferences about the whitefly host-status for four of the five plants sampled in this survey.

The five *B*. *tabaci* populations identified in this survey were all present in the sub-humid tropical agro-ecological zone. The SSA3 populations were present in the two agro-ecological zones and were collected from all five plants. These populations appeared to be adapted to the environmental conditions in the two agro-ecological zones. A preference for the sub-humid agroecological zone was, however, observed among the SSA1-SG1 and MED ASL populations. Out of the 32 times the SSA1-SG1 was identified in the survey, it only occurred once in the humid agro-ecological zone. This suggests that the Nigerian SSA1-SG1 is adapted to the sub-humid zone, which experiences less heavy and persistent rainfall.

Two samples were tested from each sampled site and a Fisher test between the results obtained from the two samples was not significant. This result reinforced our conclusion that we had identified the diversity of whiteflies in the collected samples. An increase in the number of plants and the sample locations may, perhaps, have revealed more *B*. *tabaci* diversity than is reported here. The aim of this study, however, was to identify the whitefly species colonising cassava in the region.

### *B*. *tabaci* species diversity in eastern Nigeria is lower compared to other sampled regions in sub-Saharan Africa

The SSA1-SG1 population was shown to have expanded and evolved rapidly in the Central African Republic (CAR), where 47 unique SSA1-SG1 haplotypes were identified [27]. More than half of the SSA1-SG1 populations belonged to one of the haplotypes (P18F5, GenBank code: LT707470) that, interestingly, is an identical mtCO1 sequence to the SSA1-SG1 individuals identified in this study. Tocko-Marabena *et al*. [27] identified 370 individuals belonging to this haplotype alone and reported that the haplotype occurred on all the host plants studied including *M*. *esculenta*, *Ipomoea batatas*, *Solanum melongena*, *Solanum lycopersicum*, *Arachis hypogaea*, *Sida acuta* and *Gossypium sp*. This abundant haplotype also occurred across the three agro-climatic zones surveyed by the study, which included Guinean Forest, Sudano-Guinea and Sudano Ubangian zones [27]. The tropical sub-humid and humid agro-ecological zones also extend to CAR, with the sub-humid zone occupying the upper half of CAR and the humid zone occupying the lower half [45]. In contrast, the SSA1-SG1 identified in this study were only found on cassava and *I*. *purpurea*. The Nigerian SSA1-SG1 was found predominantly in the sub-humid tropics with only one collected in the humid tropic agroecological zone. Tocko-Marabena *et al*. [27] also linked the observed population expansion of SSA1-SG1 in CAR to the upsurge of CMD in the last decade in the central African region.

The source of the introduction of the SSA1-SG1 into Nigeria or whether the population is indigenous to West Africa is not clear. Spread could have occurred by movement from the Central Africa Republic, where the haplotype P18F5 (GenBank code: LT707470) occurs in abundance. The haplotype could have also been introduced through the transport of ornamental plants to eastern Nigeria from neighbouring East African countries, although there are no records of such trades. The implications of this possible introduction are not readily evident. This survey did not, however, observe any CBSD infection nor a CMD pandemic in eastern Nigeria and there is no evidence that the SSA1-SG1-Ng occur in high abundance on cassava, as do their East and Central African (ECA) counterparts. The SSA1-SG1 has also been reported in western Nigeria by previous studies [8] but the cassava-virus pandemics and the super-abundant populations they are associated with in ECA have not been observed in the West African region. These observations could be explained by recognising that the partial mtCO1 is only a single mitochondrial barcode and does not capture the biological diversity present in the populations [3] nor the nuclear genome adaptation/evolution events, which are likely to have occurred among the Nigerian SSA1-SG1-Ng populations that prevent them from occurring at a high population density in West Africa. This suggests that the SSA1-SG1-Ng populations could have a distinct nuclear genome, different from the SSA1-SG1 population from East and Central Africa, and this possibility is currently being investigated.

Abundance data collected from 50 randomly sampled plants (S1 Table) revealed that the number of whiteflies occurring on plants in the eastern Nigeria region is low compared to the situation in East and Central Africa. Sseruwagi et al. [60] outlined a standardised method for assessing whitefly abundance, which involves counting the number of individuals on the top five leaves of each plant. They proposed this method, because previous observation had shown that the whiteflies preferred the younger leaves for feeding and oviposition. Following this protocol, only five plants, all of which were cassava, hosted more than 100 whiteflies, with only one plant hosting up to 300 individuals (S1 Table). Also, among those with less than 100 whiteflies, only five of them had more than 50 individuals and more than half of them recorded less than 30 individuals per plant. The data are in stark contrast to those recorded in East and Central Africa. For example, in a multi-year count of whitefly abundance in Uganda, there were multiple plants where more than 500 individuals were recorded on the top-five leaves of each plant [61]. Also, data presented by Alicai et al. [23] showed that the mean number of whiteflies on most of the plants they surveyed in some regions in Uganda exceeded 200. Despite these observations, however, it is important to recognise that a number of biotic and abiotic factors can influence whitefly abundance including plant age, cultivar resistance, seasonal effects and the presence or otherwise of parasitoids and natural enemies [62]. These factors could potentially contribute to maintaining lower whitefly abundance in the West African area surveyed in this study.

### Eastern Nigerian SSA1-SG1 *B*. *tabaci* populations were not “superabundant”

No evidence of CBSD infections or a CMD pandemic involving high (or “superabundant”) *B*. *tabaci* populations on cassava plants [10] was seen in our study in eastern Nigeria. Also, our field observations suggest that the West African SSA1-SG1 populations were not super-abundant in the manner of the East African populations [10,62,63], i.e. only low numbers of whiteflies per plant were seen where SSA1-SG1 *B*. *tabaci* were identified (S1 Table). This suggests that the SSA1-SG1 *B*. *tabaci* population from eastern Nigeria may be biologically different from the SSA1-SG1 in Uganda. The absence of high-density SSA1-SG1 populations and CBSD reports in eastern Nigeria is good news for cassava production in the region. The high-density SSA1-SG1 populations and the pandemics of CMD and CBSD are currently spreading westwards from East Africa, so there are genuine concerns that they may ultimately reach West Africa [26,27]. This study, therefore, provides critical and comparative baseline data that can be used to prove significantly altered epidemiologies, if or when new plant-virus pandemics and high whitefly populations arrive in eastern Nigeria.

## Conclusions

These data provide a baseline for future surveys in eastern Nigeria and are valuable for tracking future changes in *B*. *tabaci* populations in West Africa. The SSA3 *B*. *tabaci* are the predominant population in eastern Nigeria, which is a key difference to the situation in East Africa. SSA3 *B*. *tabaci* have also been reported in the West African countries of Benin and Togo, as well as in the Central African Republic [27,29]. The existence of up to 19 unique SSA3 haplotypes suggests that *B*. *tabaci* SSA3 is indigenous to West Africa. The SSA1-SG1 population did not occur in high population density in eastern Nigeria and the population consisted of only a single mtCO1 haplotype. These data show that the SSA1-SG1 may not be indigenous to the sampled region and this single haplotype may have arrived from East and Central Africa (ECA). It is also possible that the nuclear genomes of the Nigerian SSA1-SG1 and ECA SSA1-SG1 have diverged, but this cannot be determined by use of the partial mitochondrial CO1 marker.

## Supporting information

S1 Table Metadata for the first round of survey performed by J. Nwezeobi. Information is displayed for the number of whiteflies on the top five leaves of each plant surveyed.
(DOCX)

S2 Table Metadata for the second round of survey performed by the National Root Crops Research Institute. (DOCX)
