# Supplementary material for: Cassava whitefly species in eastern Nigeria and the threat of vector-borne pandemics from East and Central Africa
Source: PLoS One. 2020 May 7;15(5):e0232616. doi: 10.1371/journal.pone.0232616 (PMC7205266; doi:10.1371/journal.pone.0232616)
Supplement: S1 Table — Information is displayed for the number of whiteflies on the top five leaves of each plant surveyed. (DOCX) [file pone.0232616.s001.docx]

**S1 Table. Metadata for the First round of survey performed by J. Nwezeobi.** Information is displayed for the number of whiteflies on the top five leaves of each plant surveyed.

| **Sample Number** | **Town** | **Local Government Area** | **State** | **Longitude** | **Latitude** | **Altitude (m)** | **Whitefly species** | **Host plants** | **Agroecological zone** | **Number of whiteflies on top five leaves** |
| --- | --- | --- | --- | --- | --- | --- | --- | --- | --- | --- |
| NRI_005 | Umuokpala, Abagana | Njikoka | Anambra | 6.16840 | 6.96602 | 135.9 | SSA3 | *M. esculenta* | Sub-humid tropic | 162 |
| NRI_010 | Abagana | Njikoka | Anambra | 6.17524 | 6.97955 | 229.0 | SSA3, *Bemisia afer* | *I. purpurea* | Sub-humid tropic | 13 |
| NRI_015 | Ugbolu | Oshimili North | Delta | 6.30229 | 6.69272 | 32.3 | SSA3, SSA1-SG5 | *I. purpurea* | Sub-humid tropic | 20 |
| NRI_017 | Ugbolu | Oshimili North | Delta | 6.33321 | 6.67887 | 32.3 | SSA3 | *I. purpurea* | Sub-humid tropic | 39 |
| NRI_020 | Ugbolu | Oshimili North | Delta | 6.31430 | 6.68814 | 32.3 | SSA3 | *M. esculenta* | Sub-humid tropic | 33 |
| NRI_022 | Ngene | Oshimili North | Delta | 6.15905 | 6.74851 | 39.0 | SSA1-SG1, SSA3 | *M. esculenta* | Sub-humid tropic | 43 |
| NRI_027 | Ngene | Oshimili North | Delta | 6.11494 | 6.76503 | 43.5 | SSA3, SSA1-SG1 | *M. esculenta* | Sub-humid tropic | 58 |
| NRI_030 | Ogboji | Orumba South | Anambra | 6.02942 | 7.07838 | 300.8 | SSA3 | *M. esculenta* | Sub-humid tropic | 11 |
| NRI_034 | Ogboji | Orumba South | Anambra | 6.17892 | 6.98203 | 226.7 | SSA3 | *M. esculenta* | Sub-humid tropic | 71 |
| NRI_035 | Ogboji | Orumba South | Anambra | 6.16045 | 7.01337 | 147.3 | SSA3 | *M. esculenta* | Sub-humid tropic | 54 |
| NRI_036 | Ogboji | Orumba South | Anambra | 6.17893 | 6.99646 | 249.4 | SSA1-SG1, SSA3 | *M. esculenta* | Sub-humid tropic | 109 |
| NRI_039 | Agulu | Anaocha | Anambra | 6.17893 | 6.98203 | 226.7 | SSA1-SG5, SSA3 | *M. esculenta* | Sub-humid tropic | 7 |
| NRI_040 | Agulu | Anaocha | Anambra | 6.17892 | 6.98203 | 226.7 | SSA3 | *M. esculenta* | Sub-humid tropic | 16 |
| NRI_042 | Nkwelle Ezunanka | Oyi | Anambra | 6.20669 | 6.86384 | 133.2 | SSA1-SG1, SSA3 | *M. esculenta* | Sub-humid tropic | 21 |
| NRI_043 | Nkwelle Ezunanka | Oyi | Anambra | 6.24636 | 6.82273 | 56.1 | SSA3, SSA1-SG1 | *M. esculenta* | Sub-humid tropic | 23 |
| NRI_044 | Nsugbe | Anambra East | Anambra | 6.29905 | 6.86656 | 70.1 | SSA1-SG1, SSA3 | *M. esculenta* | Sub-humid tropic | 19 |
| NRI_045 | Nsugbe | Anambra East | Anambra | 6.29960 | 6.86609 | 70.1 | SSA1-SG1 | *M. esculenta* | Sub-humid tropic | 17 |
| NRI_046 | Nsugbe | Anambra East | Anambra | 6.29991 | 6.86581 | 70.1 | SSA1-SG1 | *M. esculenta* | Sub-humid tropic | 18 |
| NRI_047 | Umueri | Anambra East | Anambra | 6.22595 | 6.94568 | 160.3 | SSA3 | *M. esculenta* | Sub-humid tropic | 19 |
| NRI_048 | Umueri | Anambra East | Anambra | 6.22595 | 6.94571 | 160.3 | SSA3 | *M. esculenta* | Sub-humid tropic | 16 |
| NRI_051 | Awkuzu | Oyi | Anambra | 6.22481 | 6.95003 | 160.3 | SSA1-SG1, SSA3 | *M. esculenta* | Sub-humid tropic | 12 |
| NRI_050 | Aguleri | Anambra East | Anambra | 6.22528 | 6.94838 | 160.3 | SSA3, SSA1-SG1 | *M. esculenta* | Sub-humid tropic | 8 |
| NRI_052 | Awkuzu | Oyi | Anambra | 6.22487 | 6.95004 | 160.3 | SSA3 | *M. esculenta* | Sub-humid tropic | 12 |
| NRI_062 | Opi | Nsukka | Enugu | 6.85480 | 7.39390 | 436.2 | SSA3 | *M. esculenta* | Sub-humid tropic | 29 |
| NRI_065 | Opi | Nsukka | Enugu | 6.83883 | 7.37014 | 429.9 | SSA3 | *M. esculenta* | Sub-humid tropic | 33 |
| NRI_066 | Nsukka | Nsukka | Enugu | 6.63323 | 7.38167 | 422.4 | SSA3 | *R. communis* | Sub-humid tropic | 18 |
| NRI_067 | Nsukka | Nsukka | Enugu | 6.83534 | 7.36827 | 429.9 | MED-ASL, SSA3 | *M. esculenta* | Sub-humid tropic | 16 |
| NRI_072 | Nkalagu | Isienu | Ebonyi | 6.43077 | 7.49443 | 198.6 | SSA3 | *M. esculenta* | Sub-humid tropic | 19 |
| NRI_074 | Nkalagu | Isienu | Ebonyi | 6.41545 | 7.49445 | 204.6 | SSA3, SSA1-SG1 | *M. esculenta* | Sub-humid tropic | 43 |
| NRI_077 | Nkalagu | Isienu | Ebonyi | 6.46027 | 7.43090 | 349.1 | SSA3 | *M. esculenta* | Sub-humid tropic | 41 |
| NRI_079 | Nawgu | Dunukofia | Anambra | 6.32739 | 6.97631 | 58.7 | SSA3 | *M. esculenta* | Sub-humid tropic | 41 |
| NRI_080 | Nawgu | Dunukofia | Anambra | 6.32777 | 6.99444 | 70.0 | SSA1-SG1, *Bemisia afer* | *M. esculenta* | Sub-humid tropic | 38 |
| NRI_081 | Igbariam | Oyi | Anambra | 6.21913 | 7.06352 | 79.6 | SSA3 | *M. esculenta* | Sub-humid tropic | 28 |
| NRI_082 | Igbariam | Oyi | Anambra | 6.20242 | 7.00194 | 119.2 | SSA3 | *M. esculenta* | Sub-humid tropic | 31 |
| NRI_084 | Achalla | Oyi | Anambra | 6.16392 | 6.98175 | 145.0 | SSA1-SG1, *Bemisia afer* | *M. esculenta* | Sub-humid tropic | 39 |
| NRI_086 | Eziama, Obiato | Mbaitolu | Imo | 5.62020 | 6.97221 | 113.0 | SSA3 | *M. esculenta* | Sub-humid tropic | 311 |
| NRI_087 | Eziama Obiato | Mbaitolu | Imo | 5.61858 | 6.97389 | 113.0 | MED-ASL, SSA3 | *C. sativus* | Sub-humid tropic | 9 |
| NRI_088 | Eziama Obiato | Mbaitolu | Imo | 5.61592 | 6.97666 | 113.0 | SSA3 | *A. esculentus* | Sub-humid tropic | 12 |
| NRI_089 | Eziama Obiato | Mbaitolu | Imo | 5.84805 | 6.85791 | 122.3 | SSA3, SSA1-SG1 | *I. purpurea* | Sub-humid tropic | 31 |
| NRI_091 | Umunna Oha | Mbaitolu | Imo | 6.11742 | 6.83045 | 110.7 | SSA3 | *A. esculentus* | Sub-humid tropic | 43 |
| NRI_093 | Umunna Oha | Mbaitolu | Imo | 6.17889 | 6.98269 | 187.7 | SSA3, SSA1-SG1 | *M. esculenta* | Sub-humid tropic | 220 |
| NRI_094 | Okigwe | Okigwe | Abia | 5.65481 | 7.42271 | 72.3 | SSA3 | *M. esculenta* | Sub-humid tropic | 16 |
| NRI_096 | Okigwe | Okigwe | Abia | 5.57295 | 7.44921 | 101.0 | SSA3, *Bemisia afer* | *M. esculenta* | Sub-humid tropic | 260 |
| NRI_098 | Obiagu | Okigwe | Imo | 5.49043 | 7.54669 | 131.3 | MED-ASL | *A. esculentus* | Sub-humid tropic | 12 |
| NRI_101 | NRCRI Umudike | Umuahia | Abia | 6.00091 | 7.38007 | 280.7 | SSA3 | *M. esculenta* | Sub-humid tropic | 10 |
| NRI_103 | Abagana | Njikoka | Anambra | 6.17979 | 6.97620 | 202.0 | SSA3 | *M. esculenta* | Sub-humid tropic | 51 |
| NRI_104 | Abagana | Njikoka | Anambra | 6.17991 | 6.97549 | 202.0 | SSA1-SG5, SSA3 | *M. esculenta* | Sub-humid tropic | 48 |
| NRI_105 | Anwai | Oshimili North | Delta | 6.23163 | 6.70295 | 43.9 | SSA1-SG1, SSA3 | *M. esculenta* | Sub-humid tropic | 46 |
| NRI_107 | Ngene | Oshimili North | Delta | 6.16197 | 6.74847 | 32.8 | SSA3 | *M. esculenta* | Sub-humid tropic | 72 |
| NRI_108 | Ugbolu | Oshimili North | Delta | 6.11436 | 6.76466 | 27.7 | SSA3 | *I. purpurea* | Sub-humid tropic | 39 |
