# Supplementary material for: Cassava whitefly species in eastern Nigeria and the threat of vector-borne pandemics from East and Central Africa
Source: PLoS One. 2020 May 7;15(5):e0232616. doi: 10.1371/journal.pone.0232616 (PMC7205266; doi:10.1371/journal.pone.0232616)
Supplement: S2 Table — (DOCX) [file pone.0232616.s002.docx]

S2 Table. Metadata for the second round of survey performed by the National Root Crops Research Institute.

| **Sample Number** | **Town** | **Local Government Area** | **State** | **Longitude** | **Latitude** | **Altitude (m)** | **Whitefly species** | **Host plants** | **Agroecological zone** |
| --- | --- | --- | --- | --- | --- | --- | --- | --- | --- |
| NRCRI_01 | Umuawa | Umuahia South | Abia | 5.56284 | 7.48056 | 110.0 | SSA3 | *M. esculenta* | Sub-humid tropic |
| NRCRI_02 | umuohiri Akoliemenyi | Bende | Abia | 5.66476 | 7.55749 | 103.0 | SSA3 | *M. esculenta* | Sub-humid tropic |
| NRCRI_03 | Ibina ukwu, Igbere | Bende | Abia | 5.73369 | 7.63556 | 206.0 | SSA3 | *M. esculenta* | Sub-humid tropic |
| NRCRI_04 | Nguzu junction | Ohafia | Abia | 5.73585 | 5.73585 | 135.0 | SSA3 | *M. esculenta* | Sub-humid tropic |
| NRCRI_05 | Amakama | Umuahia South | Abia | 5.45648 | 7.48023 | 145.0 | SSA1-SG5, SSA3 | *M. esculenta* | Sub-humid tropic |
| NRCRI_06 | Aba road | Isiala Ngwa South | Abia | 5.29550 | 7.48408 | 116.0 | SSA1-SG5, SSA3 | *M. esculenta* | Humid tropic |
| NRCRI_07 | Obegu town | Ugwunbo | Abia | 4.98041 | 7.31992 | 51.0 | SSA3, SSA1-SG5 | *M. esculenta* | Humid tropic |
| NRCRI_08 | Ikpokwu Asa | Ukwa West | Abia | 5.92991 | 7.27453 | 38.0 | SSA3 | *M. esculenta* | Sub-humid tropic |
| NRCRI_09 | Mgbelu Umunnekwu | Isuikwuato | Abia | 5.46541 | 7.38722 | 265.0 | SSA1-SG5, SSA3 | *M. esculenta* | Sub-humid tropic |
| NRCRI_10 | Isiaka | Ivo | Abia | 5.86571 | 7.53755 | 77.0 | *Bemisia afer*, SSA3 | *M. esculenta* | Sub-humid tropic |
| NRCRI_11 | Agalagu Amachi | Abakaliki | Ebonyi | 6.29875 | 8.17218 | 43.0 | SSA3 | *M. esculenta* | Sub-humid tropic |
| NRCRI_12 | Akalegu | Ebonyi | Ebonyi | 6.35652 | 8.05231 | 62.0 | SSA3 | *M. esculenta* | Sub-humid tropic |
| NRCRI_13 | Umuebe | Ohaukwu | Ebonyi | 6.37613 | 7.48056 | 93.0 | SSA3, SSA1-SG1 | *M. esculenta* | Sub-humid tropic |
| NRCRI_14 | Ngbo | Ohaukwu | Ebonyi | 6.58765 | 8.02075 | 93.0 | SSA3 | *M. esculenta* | Sub-humid tropic |
| NRCRI_15 | Ebonyi/Enugu Boundary | Nkanu East | Ebonyi | 6.46574 | 7.73291 | 87.0 | SSA1-SG5, SSA3 | *M. esculenta* | Sub-humid tropic |
| NRCRI_16 | Amonu Ngwo | Udi | Enugu | 6.44470 | 7.42688 | 388.0 | SSA3, SSA1-SG1 | *M. esculenta* | Sub-humid tropic |
| NRCRI_17 | Umurumbe | Udi | Enugu | 6.59058 | 7.41790 | 442.0 | SSA1-SG5, SSA3 | *M. esculenta* | Sub-humid tropic |
| NRCRI_18 | Diogbe | Igbo-Etiti | Enugu | 6.68437 | 7.41666 | 474.0 | SSA3, SSA1-SG1 | *M. esculenta* | Sub-humid tropic |
| NRCRI_19 | Iheaka | Igbeze South | Enugu | 6.90176 | 7.47071 | 445.0 | SSA1-SG1, SSA3 | *M. esculenta* | Sub-humid tropic |
| NRCRI_20 | Umuogbo Inyi | Igboeze North | Enugu | 6.97069 | 7.53615 | 461.0 | SSA3 | *M. esculenta* | Sub-humid tropic |
| NRCRI_21 | Obioma | Udi | Enugu | 6.36514 | 7.39224 | 416.0 | SSA1-SG5, *Bemisia afer* | *M. esculenta* | Sub-humid tropic |
| NRCRI_22 | Eluama Abueke | Ihitte Uboma | Enugu | 5.61375 | 7.39224 | 140.0 | SSA1-SG1, SSA3 | *M. esculenta* | Sub-humid tropic |
| NRCRI_23 | Nkumeato | Ihitte Uboma | Imo | 5.61165 | 7.33233 | 410.0 | SSA3 | *M. esculenta* | Sub-humid tropic |
| NRCRI_24 | Umuohu Mpam | Ahiazu Mbaise | Imo | 5.58946 | 7.28428 | 410.0 | SSA3 | *M. esculenta* | Sub-humid tropic |
| NRCRI_25 | Okoroukwu Ezuhu | Aboh Mbaise | Imo | 5.48216 | 7.26996 | 134.0 | SSA3 | *M. esculenta* | Sub-humid tropic |
| NRCRI_27 | Egbelubi | Ngor Okpala | Imo | 5.30360 | 7.25677 | 88.0 | SSA3 | *M. esculenta* | Sub-humid tropic |
| NRCRI_30 | Oguta | Oguta | Imo | 5.69404 | 6.78728 | 19.0 | SSA3 | *M. esculenta* | Sub-humid tropic |
| NRCRI_31 | Otulu | Oru West | Imo | 5.70280 | 6.91327 | 72.0 | SSA3 | *M. esculenta* | Sub-humid tropic |
| NRCRI_32 | Umuduru Umuelemai | Isiala Mbano | Imo | 5.69072 | 7.23957 | 165.0 | SSA3 | *M. esculenta* | Sub-humid tropic |
| NRCRI_33 | Ezioha Mgbowo | Awgu | Enugu | 6.05580 | 7.29206 | 56.0 | SSA3 | *M. esculenta* | Sub-humid tropic |
| NRCRI_36 | Nibo-Nise | Awka south | Anambra | 6.17879 | 7.04294 | 118.0 | SSA3, SSA1-SG1 | *M. esculenta* | Sub-humid tropic |
| NRCRI_39 | Isuaniocha | Awka North | Anambra | 6.24863 | 7.05323 | 44.0 | SSA3, *Bemisia afer* | *M. esculenta* | Sub-humid tropic |
| NRCRI_43 | Obosi | Idemmili North | Anambra | 6.12728 | 6.83489 | 58.0 | SSA3, SSA1-SG1 | *M. esculenta* | Sub-humid tropic |
| NRCRI_45 | Uli | Ihiala | Anambra | 5.78099 | 6.85866 | 76.0 | SSA3, SSA1-SG1 | *M. esculenta* | Sub-humid tropic |
| NRCRI_46 | Ugwu-Oboshi-Ibusa | Oshimili | Delta | 6.16922 | 6.60212 | 23.0 | SSA1-SG5, SSA1-SG1 | *M. esculenta* | Sub-humid tropic |
| NRCRI_48 | Ogwashi Olor | Aniocha South | Delta | 6.10261 | 6.52338 | 71.0 | *Bemisia afer*, SSA3 | *M. esculenta* | Sub-humid tropic |
| NRCRI_50 | Umu-Osummili-Ossissa | Ndokwu East | Delta | 5.91756 | 6.47882 | 26.0 | SSA1-SG1 | *M. esculenta* | Sub-humid tropic |
| NRCRI_51 | Ofagbe | Isoko North | Delta | 5.52246 | 6.33790 | 22.0 | SSA1-SG5, SSA3 | *M. esculenta* | Sub-humid tropic |
| NRCRI_52 | Emede | Isoko South | Delta | 5.43092 | 6.18429 | 18.0 | SSA3 | *M. esculenta* | Sub-humid tropic |
| NRCRI_53 | Agadama | Ughelli North | Delta | 5.23656 | 6.04095 | 15.0 | SSA3 | *M. esculenta* | Humid tropic |
| NRCRI_54 | Abaraka | Etiop East | Delta | 5.84184 | 6.09508 | 36.0 | SSA3 | *M. esculenta* | Sub-humid tropic |
| NRCRI_55 | Amudemi Amundemi | Okpe | Delta | 5.66246 | 5.77330 | 11.0 | SSA3 | *M. esculenta* | Sub-humid tropic |
| NRCRI_56 | Alifekede Agbor | Ika | Delta | 6.27112 | 6.10304 | 166.0 | SSA1-SG5, SSA3 | *M. esculenta* | Sub-humid tropic |
| NRCRI_61 | Ekperi | Etsako Central | Edo | 7.02849 | 6.40633 | 88.0 | SSA1-SG5, SSA1-SG1 | *M. esculenta* | Sub-humid tropic |
| NRCRI_62 | Iviokpopi-Ivioghe Agenebode | Etsako East | Edo | 7.12103 | 6.64701 | 179.0 | SSA3 | *M. esculenta* | Sub-humid tropic |
| NRCRI_63 | Afashio Uzaire | Etsako West | Edo | 7.09584 | 6.32423 | 228.0 | SSA1-SG5 | *M. esculenta* | Sub-humid tropic |
| NRCRI_64 | Ojavun | Owan East | Edo | 6.93466 | 8.97166 | 87.0 | SSA1-SG1, SSA1-SG5 | *M. esculenta* | Sub-humid tropic |
| NRCRI_65 | Sabongida | Owan West | Edo | 6.89565 | 5.92001 | 72.0 | SSA3, SSA1-SG5 | *M. esculenta* | Sub-humid tropic |
| NRCRI_66 | Borobara | Tai | Rivers | 4.72596 | 7.23927 | 20.0 | SSA1-SG5 | *M. esculenta* | Humid tropic |
| NRCRI_67 | Bori | Tai | Rivers | 4.73457 | 7.34657 | 18.0 | SSA1-SG5, SSA3 | *M. esculenta* | Humid tropic |
| NRCRI_68 | Isiodu | Emohua | Rivers | 4.88767 | 6.86175 | 18.0 | SSA3 | *M. esculenta* | Humid tropic |
| NRCRI_69 | Ibaa | Emohua | Rivers | 4.94825 | 6.79856 | 14.0 | SSA3 | *M. esculenta* | Humid tropic |
| NRCRI_70 | Elele | Elele | Rivers | 5.07089 | 6.75580 | 31.0 | SSA3 | *M. esculenta* | Humid tropic |
| NRCRI_72 | Omerelu | Ikwerre | Rivers | 5.13467 | 6.83693 | 30.0 | SSA3 | *M. esculenta* | Humid tropic |
| NRCRI_73 | Ogoja | Ogoja | Cross River | 6.65316 | 8.82272 | 66.0 | SSA3 | *M. esculenta* | Sub-humid tropic |
| NRCRI_74 | Gakem | Bekwara | Cross River | 6.77978 | 8.99800 | 158.0 | SSA3 | *M. esculenta* | Sub-humid tropic |
| NRCRI_75 | Benue/Cross-River boundary | Obudu | Cross River | 6.68506 | 9.15223 | 227.0 | SSA3, SSA1-SG5 | *M. esculenta* | Sub-humid tropic |
| NRCRI_76 | Ajassor | Etung | Cross River | 5.86900 | 8.81291 | 126.0 | *Bemisia afer,* SSA3 | *M. esculenta* | Humid tropic |
| NRCRI_77 | Mfom | Etung | Cross River | 5.81330 | 8.84527 | 75.0 | SSA3, *Bemisia afer* | *M. esculenta* | Humid tropic |
| NRCRI_71 | Imiringi | Ogbia | Bayelsa | 4.86862 | 6.37601 | -33.0 | SSA3, SSA1-SG1 | *M. esculenta* | Humid tropic |
| NRCRI_79 | Adun | Obubra | Cross River | 5.96374 | 8.25976 | 73.0 | SSA3 | *M. esculenta* | Sub-humid tropic |
| NRCRI_80 | Akamkpa | Akamkpa | Cross River | 5.41683 | 8.19876 | 139.0 | SSA3 | *M. esculenta* | Humid tropic |
| NRCRI_81 | Akantri | Akamkpa | Cross River | 5.16355 | 8.53448 | 76.0 | SSA3 | *M. esculenta* | Humid tropic |
| NRCRI_82 | Odukpani Jxtn | Odukpani | Cross River | 5.16639 | 8.32689 | 14.0 | SSA3 | *M. esculenta* | Humid tropic |
| NRCRI_83 | Nsisat 11 | Odukpani | Cross River | 5.17264 | 8.13441 | 74.0 | SSA3 | *M. esculenta* | Humid tropic |
| NRCRI_84 | Ntondang | Obot Akara | Akwa Ibom | 5.48881 | 7.54309 | 70.0 | SSA3 | *M. esculenta* | Sub-humid tropic |
| NRCRI_85 | Edieme | Ikono | Akwa Ibom | 5.20147 | 7.80877 | 68.0 | SSA3 | *M. esculenta* | Humid tropic |
| NRCRI_86 | Abak Usong Idim | Abak | Akwa Ibom | 4.95588 | 7.79549 | 20.0 | SSA3, *Bemisia afer* | *M. esculenta* | Humid tropic |
| NRCRI_87 | Ikot Etiti | Mkpat Enin | Akwa Ibom | 4.61817 | 7.73592 | 20.0 | SSA3, *Bemisia afer* | *M. esculenta* | Humid tropic |
